# Dynamic frailty trajectories and risk of age-related macular degeneration: a prospective cohort study in UK Biobank

**DOI:** 10.1007/s11357-025-02066-x

**Published:** 2026-01-02

**Authors:** Yu Peng, Yuzhou Zhang, Ka Wai Kam, Mary Ho, Simon Sezto, Sunny Au, Charlene C. Yim, Xiujuan Zhang, Mandy P. H. Ng, Patrick Ip, Alvin Young, Chi Pui Pang, Clement C. Tham, Li Jia Chen, Jason C. Yam

**Affiliations:** 1 Department of Ophthalmology and Visual Sciences, The Chinese University of Hong Kong, Hong Kong SAR, China; 2 Department of Ophthalmology and Visual Sciences, Prince of Wales Hospital, Hong Kong Eye Hospital, Hong Kong SAR, China; 3 Department of Ophthalmology, Tung Wah Eastern Hospital, Hong Kong SAR, China; 4 Department of Ophthalmology, The University of Hong Kong, Hong Kong SAR, China; 5 Department of Paediatrics and Adolescent Medicine, Li Ka Shing Faculty of Medicine, The University of Hong Kong, Hong Kong SAR, China; 6 Hong Kong Hub of Paediatric Excellence, The Chinese University of Hong Kong, Hong Kong SAR, China; 7 Shantou University, The Chinese University of Hong Kong Joint Shantou International Eye Centre, Shantou, China; 8 Hong Kong Eye Hospital, Hong Kong SAR, China; 9 Department of Ophthalmology, Hong Kong Children’s Hospital, Hong Kong SAR, China

**Keywords:** Frailty, Trajectories, Age-related macular degeneration, Aging, UK Biobank

## Abstract

Age-related macular degeneration (AMD) is a leading cause of irreversible vision loss in the aging population. Although frailty has been recognized as a potential risk factor, previous studies relying on static assessments cannot capture its dynamic nature. This study aims to prospectively evaluate the association between dynamic frailty trajectories and incident AMD. The baseline cohort included 462,573 UK Biobank participants who were free of AMD and had frailty phenotype (FP) data at enrollment. Among them, 53,059 with at least one follow-up FP assessment comprised the trajectory cohort. Participants were categorized as nonfrail, prefrail, or frail based on their FP scores. Annualized frailty progression (ΔFP/year) was estimated via linear regression. Cox models assessed hazard ratios (HRs) and 95% confidence intervals (CIs) for AMD. During a median follow-up of 11.8 years in the baseline cohort, 13,428 incident AMD cases (2.9%) were documented. Prefrail (HR:1.14; 95% CI:1.10–1.18) and frail (HR:1.36; 95% CI: 1.26–1.47) individuals had significantly higher AMD risks compared to nonfrail participants. In the frailty trajectory analysis with a median follow-up duration of 3.3 years, 852 incident AMD cases (1.6%) were recorded. Each 1-point FP increase conferred 28% higher AMD risk (HR: 1.28; 95% CI: 1.18–1.38), while each 0.1-point/year ΔFP increase independently elevated risk by 15% (HR: 1.15; 95% CI: 1.10–1.21). Compared to stable nonfrail, prefrailty aggravation showed highest AMD risk (HR: 2.26; 95% CI: 1.51–3.37), followed by frailty alleviation (HR: 1.73; 95% CI: 0.98–3.04) and frailty maintenance (HR: 1.72; 95% CI: 0.86–3.41). Progressive frailty trajectories, independent of baseline status, is associated with increased incident AMD risk. Early interventions targeting frailty progression may mitigate AMD risk in aging populations.

**Supplementary Information:**

The online version contains supplementary material available at https://doi.org/10.1007/s11357-025-02066-x.

## Introduction

Age-related macular degeneration (AMD) is the predominant cause of irreversible vision impairment among elderly populations in industrialized nations [1]. Epidemiological projections estimated that approximately 288 million individuals worldwide will be affected by AMD by 2040, driven by the global aging population [2]. Established risk factors for AMD include advanced age, genetic susceptibility, tobacco use, and cardiovascular comorbidities [3–5]. Emerging evidence further suggests that systemic health deterioration, such as frailty status, may play an overlooked role in the development of AMD [6].

Frailty, recognized as a clinical indicator of accelerated biological aging, manifests as a multisystem syndrome characterized by diminished physiological reserves, impaired stress response capacity, and functional deterioration [7, 8]. The two most widely adopted frailty assessment tools are the physical frailty phenotype (FP) and the frailty index (FI). FP defines a clinical syndrome based on five criteria, including weight loss, self-reported exhaustion, decreased grip strength, slower gait speed, and reduced physical activity, while FI measures the accumulation of health deficits [9, 10]. This geriatric syndrome has emerged as a public health challenge, demonstrating established associations with neurodegenerative disorders [11, 12], metabolic dysfunction [13], cardiovascular diseases [14] and all-cause mortality [15].

Both frailty and AMD are associated with senescence processes, potentially converging through shared pathophysiological pathways, such as chronic low-grade inflammation [16, 17], oxidative stress [18, 19], mitochondrial dysfunction [20, 21], and dysregulated complement activation [22, 23]. While a previous study suggested an association between frailty and increased AMD risk [6], gaps persist in understanding the longitudinal dynamics of this relationship. Specifically, no evidence exists on how temporal changes in frailty trajectories, such as aggravation, maintenance, or alleviation, influence AMD incidence. This represents a limitation given frailty's dynamic nature and potential reversibility through clinical or behavioral interventions [24, 25]. Current dependence on single-timepoint assessments overlooks the dynamic biological processes reflected in longitudinal trajectory patterns, thus potentially limiting clinical applicability.

Given this background, we hypothesize that progressive frailty trajectories may exhibit a dose–response relationship with AMD risk. Therefore, this study aimed to investigate the associations between dynamic frailty progression and incident AMD, and quantitatively assess the impacts of distinct frailty trajectory patterns on AMD development.

## Methods

### Study design and participants

The UK Biobank is a prospective population-based cohort study that recruited over 500,000 participants aged 37–73 years from 22 assessment centers across the United Kingdom between 2006 and 2010. The study’s design, protocols, and methodology have been described in previous studies [26, 27]. To date, longitudinal follow-up assessments have been conducted in three phases: the first (2012–2013), the second (ongoing since 2014), and the third (initiated in 2019). At baseline and all follow-up visits, participants underwent standardized physical examinations and provided sociodemographic and lifestyle information through touchscreen questionnaires. Clinical assessments were linked to national electronic health records, including primary care datasets, hospital inpatient admissions, cancer registries, and mortality records. Ethical approval for the UK Biobank was obtained from the North West Multi-Center Research Ethics Committee (21/NW/0157). This study utilized UK Biobank data under the application number 91320.

In this study, from the baseline cohort of 502,308 participants, we excluded individuals with pre-existing AMD or incomplete FP and covariate data, resulting in 462,573 eligible participants for baseline analyses. Among them, 53,059 participants who had at least one follow-up assessment for FP and were AMD-free before the trajectories assessment were included in the trajectories analysis. Participants from the third assessment phase were excluded due to limited sample size (approximately 6,000) and short follow-up duration. Moreover, 458,224 and 55,827 participants with full FI data at baseline and who completed the initial or subsequent repeated assessments were involved in the sensitivity analyses. Details of the study design and sample selection are shown in Supplementary Figs. 1 and 2.

### Assessment of AMD

AMD cases were identified through: (1) electronic health records using International Classification of Diseases (ICD) codes (ICD-10: H35.3; ICD-9: 362.5), (2) structured interviews administered by trained clinical staff (field code 20,002 [1528]), and (3) self-reported touchscreen questionnaire data (field codes 6148 and 5923) [4].

### Assessment of frailty

The classification of FP, originally developed by Fried et al. in 2001 [9], was adapted within the UK Biobank framework as described in previous studies [28]. FP assessment involves 5 criteria: weight loss, exhaustion, grip strength weakness, slow walking speed, and low physical activity. Detailed definitions for each criterion are provided in Supplementary Table 1. The severity of frailty is determined by the number of criteria met, with scores ranging from 0 to 5. Consistent with previous studies [29, 30], participants were categorized into three groups based on their FP scores: nonfrailty (FP score: 0), prefrailty (FP score: 1 and 2), and frailty (FP score: 3, 4 and 5).

The FI approach, developed by Rockwood and Mitnitski, quantifies health deficits across multiple domains [10, 31]. A validated 49-item FI [32] was constructed using UK Biobank data spanning self-reported health status, comorbidities, functional disabilities, and psychological symptoms (Supplementary Table 2). The FI score was calculated as the proportion of deficits present relative to the total assessed (range: 0–1), with higher values indicating greater frailty. Participants with missing data for ≥ 10 items were excluded. Individuals were classified into three categories based on their FI scores: nonfrailty (FI ≤ 0.10), prefrailty (0.10 < FI ≤ 0.21), and frailty (FI > 0.21) [32].

The FP was prioritized as the primary exposure variable due to its direct alignment with clinical definitions of physical frailty and its utility in guiding preventive interventions. In contrast, the FI, which captures multidimensional health deficits, was incorporated into sensitivity analyses to evaluate robustness and address potential confounding from heterogeneous frailty manifestations.

### Assessment of covariates

Covariates included age, sex, ethnicity, Townsend deprivation index, education, body mass index (BMI), alcohol consumption, smoking status, hypertension, and diabetes. The detailed definitions of each variable are shown in Supplementary Table 3.

### Statistical analysis

Cox proportional hazards regression models were used in both baseline and trajectory analyses to estimate hazard ratios (HRs) and 95% confidence intervals (CIs) for the associations between frailty and incident AMD. Follow-up duration was defined as the interval from the baseline assessment (for baseline analysis) or the final follow-up assessment (for trajectory analysis) to the occurrence of AMD diagnosis, death, loss to follow-up, or the censoring (December 31, 2020, for England/Wales; January 18, 2021, for Scotland), which was the earliest. Three models were constructed: Model 1 adjusted for age and sex; Model 2 further adjusted for ethnicity, educational attainment, BMI, Townsend deprivation index, smoking status and alcohol consumption; and Model 3 additionally adjusted for hypertension and diabetes.

Frailty progression rates were assessed using linear regression, with FP (range: 0–5) and FI (range: 0–1) scores treated as continuous dependent variables and follow-up time as the independent variable. Annualized rates of frailty progression (ΔFP: points/year; ΔFI: points/year) were derived from regression coefficients, incorporating all available longitudinal assessments. Nonlinear associations between annualized rates of frailty progression and AMD risk were evaluated using restricted cubic spline (RCS) models with three knots.

To capture dynamic frailty changes beyond static baseline assessments, transitions between defined frailty states (nonfrail, prefrail, and frail) from baseline to final assessment were characterized as trajectories and examined for their association with incident AMD development. Participants were categorized into seven groups: nonfrailty maintenance, nonfrailty aggravation, prefrailty alleviation, prefrailty maintenance, prefrailty aggravation, frailty alleviation, and frailty maintenance, with nonfrailty maintenance being the reference group [33]. Population-attributable risk (PAR) was estimated to quantify the hypothetical reduction in AMD incidence achievable by mitigating frailty progression in nonfrail and prefrail subgroups or stabilizing frailty status in frail populations.

Stratified analyses were conducted across subgroups defined by age (< 60 and ≥ 60 years), sex, smoking status (current and former/never), alcohol consumption (current and former/never), hypertension, and diabetes. Interaction effects were tested via likelihood ratio tests comparing models with and without multiplicative interaction terms.

We conducted a series of sensitivity analyses to evaluate the robustness of the findings. First, the FI was used instead of the FP to reassess frailty status and examine the associations between frailty and incident AMD. Second, individuals with less than 2 years of follow-up were excluded to minimize potential bias from reverse causality. Third, participants reporting poor overall health at baseline were removed to reduce the influence of baseline health status on frailty classification. Fourth, additional adjustments were made for plasma high-density lipoprotein (HDL) cholesterol, low-density lipoprotein (LDL) cholesterol, triglyceride levels, and the AMD polygenic risk score to account for confounding effects of metabolic factors and genetic susceptibility. Finally, the covariates estimated in the final assessment were used instead of those at baseline in the multivariable model.

All statistical analyses were performed using R software version 4.3.1. Statistical significance was defined as a two-tailed P-value below 0.05.

## Results

### Participant characteristics

The baseline analysis included 462,573 participants (mean age: 56.4 ± 8.1 years; 45.3% male), with 55.1%, 40.8%, and 4.0% classified into nonfrailty, prefrailty, and frailty groups, respectively. A subset of 53,059 participants was included in trajectory analysis, with baseline frailty distributions of 63.8% (nonfrailty), 34.6% (prefrailty), and 1.6% (frailty). After a median follow-up of 3.3 years, these proportions shifted to 42.6%, 54.3%, and 3.1%, respectively (Table 1 and Supplementary Table 4). Prefrail and frail individuals, compared to nonfrail participants, were more likely to be older, of non-white ethnicity, nondrinkers, current smokers, have lower educational attainment and socioeconomic status, and also exhibited higher BMI and greater prevalence of diabetes and hypertension.

**Table 1:** Baseline characteristics of participants in the baseline and the trajectories assessment according to frailty phenotype

	Baseline assessment^b^			Trajectories assessment^c^		
Characteristics^a^	Nonfrailty	Prefrailty	Frailty	Nonfrailty	Prefrailty	Frailty
Sample size, *n*	255,079	188,948	18,546	22,628	28,801	1630
Age, mean (SD), years	55.97 ± 8.1	56.80 ± 8.1	57.75 ± 7.7	54.48 ± 7.4	56.13 ± 7.6	56.66 ± 7.8
Men, n (%)	123,097 (48.3)	79,906 (42.3)	6724 (36.3)	8542 (37.7)	16,400 (56.9)	725 (44.5)
White ethnicity, *n* (%)	245,853 (96.4)	176,031 (93.2)	16,414 (88.5)	22,077 (97.6)	27,896 (96.9)	1544 (94.7)
University or college degree, *n* (%)	95,227 (37.3)	54,949 (29.1)	3121 (16.8)	11,047 (48.8)	12,556 (43.6)	491 (30.1)
Townsend deprivation index, mean (SD)	− 1.72 ± 2.85	− 1.05 ± 3.17	0.41 ± 3.56	− 2.02 ± 2.67	− 1.89 ± 2.72	− 0.95 ± 3.21
Drinking status, *n* (%)						
Current	241,589 (94.7)	171,198 (90.6)	14,699 (79.3)	21,674 (95.8)	27,376 (95.0)	1447 (88.8)
Former	6009 (2.4)	7941 (4.2)	1889 (10.2)	419 (1.9)	666 (2.3)	98 (6.0)
Never	7481 (2.9)	9809 (5.2)	1958 (10.6)	535 (2.4)	761 (2.6)	85 (5.2)
Smoking status, *n* (%)						
Current	22,277 (8.7)	21,692 (11.5)	3505 (18.9)	1239 (5.5)	1958 (6.8)	181 (11.1)
Former	87,698 (34.4)	65,888 (34.9)	6391 (34.5)	7202 (31.8)	9861 (34.2)	599 (36.7)
Never	145,104 (56.9)	101,368 (53.6)	8650 (46.6)	14,187 (62.7)	16,984 (59.0)	850 (52.1)
Body mass index, mean (SD), kg/m^2^	26.50 ± 4.08	28.18 ± 5.01	31.07 ± 6.55	25.65 ± 4.00	27.28 ± 4.17	30.73 ± 6.27
Diabetes, n (%)	6,483 (2.5)	12,094 (6.4)	3102 (16.7)	352 (1.6)	1091 (3.8)	196 (12.0)
Hypertension, *n* (%)	54,714 (21.4)	56,997 (30.2)	8591 (46.3)	3941 (17.4)	7402 (25.7)	655 (40.2)

^a^Characteristics are presented for all participants at the time of their enrollment

^b^Baseline assessment refers to the single-timepoint frailty measurement at enrollment

^c^Trajectories assessment refers to the longitudinal analysis of frailty changes across follow-up measurements in the subcohort with repeated data

### Associations between baseline assessment of frailty and incident AMD risk

Over a median follow-up of 11.8 years from the baseline assessment, 13,428 incident AMD cases (2.9%) were identified out of 462,573 participants. Compared to nonfrail participants, prefrail and frail individuals demonstrated significantly elevated AMD risks, with adjusted HRs of 1.14 (95% CI: 1.10–1.18) and 1.36 (95% CI: 1.26–1.47), respectively, after full adjustment for covariates in Model 3 (Table 2). Each 1-point increase in frailty score was associated with a 10% higher AMD risk (HR: 1.10; 95% CI: 1.08–1.13). Analysis of frailty components indicated associations between AMD risk and weight loss, exhaustion, weak grip strength, and slow walking speed, while no significant association was observed for low physical activity (Supplementary Table 5).

**Table 2:** Associations of baseline and long-term changes in frailty with incident AMD according to frailty phenotype

Frailty phenotype	Cases/participants	Absolute risk, %	HR, 95% CI		
			Model 1	Model 2	Model 3
Baseline assessment^a^					
Nonfrailty	6471/255,079	2.54	1 (Reference)	1 (Reference)	1 (Reference)
Prefrailty	6129/188,948	3.24	1.23 (1.18, 1.27)	1.18 (1.13, 1.22)	1.14 (1.10, 1.18)
Frailty	828/18,546	4.46	1.68 (1.57, 1.81)	1.50 (1.39, 1.62)	1.36 (1.26, 1.47)
Per 1-point increase			1.18 (1.15, 1.20)	1.14 (1.12, 1.16)	1.10 (1.08, 1.13)
Trajectories assessment^b^					
Nonfrailty	303/22,628	1.34	1 (Reference)	1 (Reference)	1 (Reference)
Prefrailty	499/28,801	1.73	1.48 (1.28, 1.72)	1.48 (1.27, 1.71)	1.43 (1.24, 1.66)
Frailty	50/1630	3.07	2.57 (1.91, 3.47)	2.60 (1.90, 3.56)	2.31 (1.68, 3.17)
Per 1-point increase			1.31 (1.22, 1.41)	1.31 (1.22, 1.42)	1.28 (1.18, 1.38)
ΔFP per 0.1-point/year increase			1.16 (1.11, 1.21)	1.16 (1.11, 1.21)	1.15 (1.10, 1.21)

Model 1: adjusted for age and sex

Model 2: Model 1 variables plus ethnicity, Townsend deprivation index, education, body mass index, alcohol consumption, and smoking status

Model 3: Model 2 variables plus diabetes, and hypertension

For trajectory analysis in frailty (ΔFP), the baseline frailty score was further adjusted in the Cox regression models

^a^Baseline assessment refers to the single-timepoint frailty measurement at enrollment

^b^Trajectories assessment refers to the longitudinal analysis of frailty changes across follow-up measurements in the subcohort with repeated data

*AMD* age-related macular degeneration, *FP* frailty phenotype, *HR* hazard ratio, *CI* confidence interval

### Associations between trajectory assessment of frailty and incident AMD risk

In the trajectory analysis with a median follow-up of 3.3 years, 852 AMD cases (1.6%) were documented out of 53,059 participants. Compared to the nonfrailty group, prefrail and frail participants at final assessment exhibited HRs of 1.43 (95% CI: 1.24–1.66) and 2.31 (95% CI: 1.68–3.17), respectively, after full adjustment (Table 2). Each 1-point increase in frailty score was correlated with a 28% elevated AMD risk (HR: 1.28; 95% CI: 1.18–1.38). Furthermore, a 0.1-point/year increase in frailty progression (ΔFP) was associated with a 15% higher AMD risk (HR: 1.15; 95% CI: 1.10–1.21) after adjusting for baseline FP. RCS analysis confirmed a linear dose–response relationship between ΔFP and AMD risk (*P*-overall < 0.001; *P*-nonlinear = 0.085; Supplementary Fig. 3).

### Stratified analyses

**Fig. 1:**
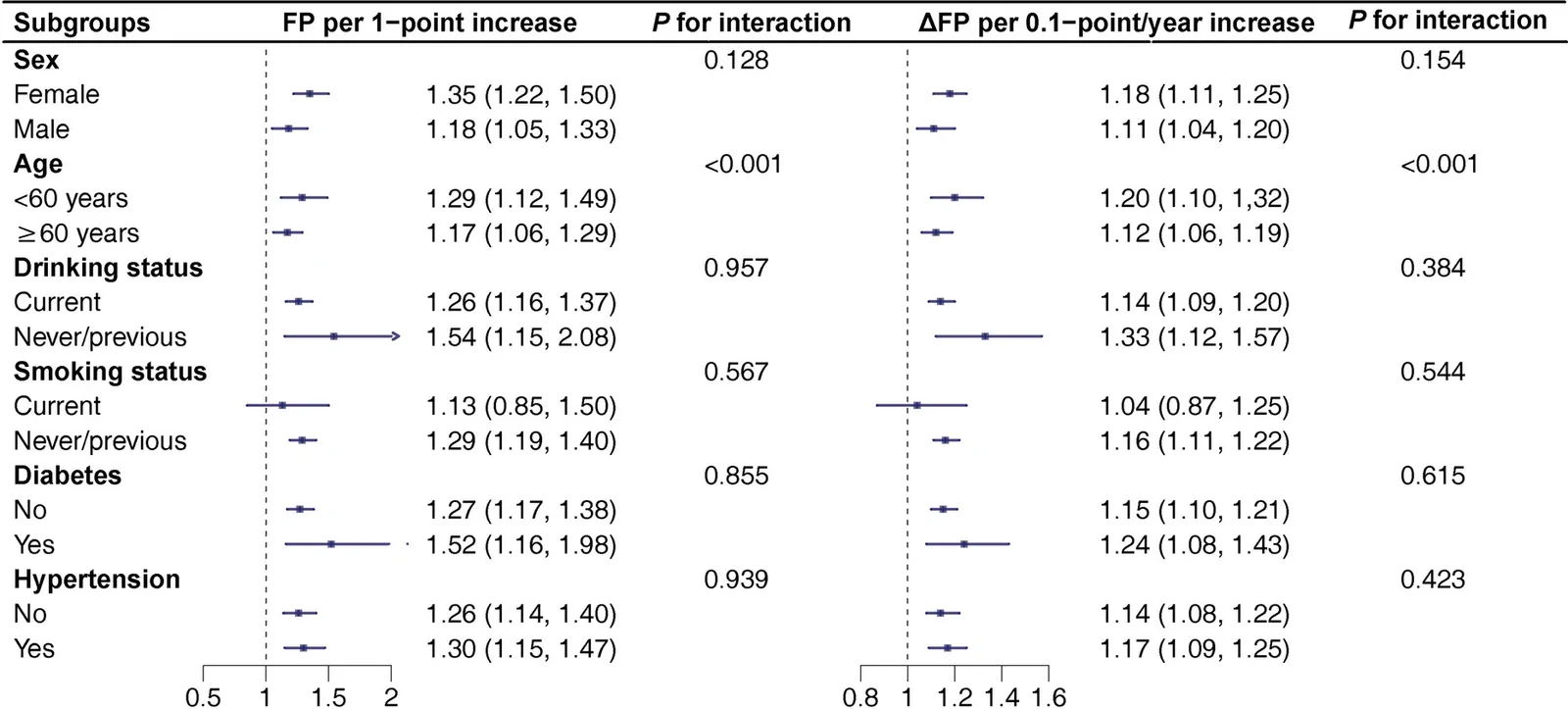
Stratification analyses for associations of long-term frailty changes with incident AMD. Results are present as hazard ratios (95% confidence intervals). Models were adjusted for age, sex, ethnicity, Townsend deprivation index, education, body mass index, alcohol consumption, smoking status, diabetes, and hypertension. For the trajectory analysis of frailty (ΔFP), the baseline frailty phenotype score was further adjusted in the Cox regression models. The stratification factors were removed from the corresponding models. AMD, age-related macular degeneration; FP, frailty phenotype

Stratified analyses revealed significant effect modification by age (*P*-interaction < 0.001). Participants aged < 60 years demonstrated increased AMD risks with each 1-point increase in FP (HR: 1.29; 95% CI: 1.12–1.49) or a 0.1-point/year rise in ΔFP (HR: 1.20; 95% CI: 1.10–1.32; Fig. 1). No significant interactions were observed across sex, smoking status, alcohol consumption, diabetes, or hypertension subgroups (all *P*-interaction > 0.05).

### Associations of frailty trajectories with incident AMD

**Fig. 2:**
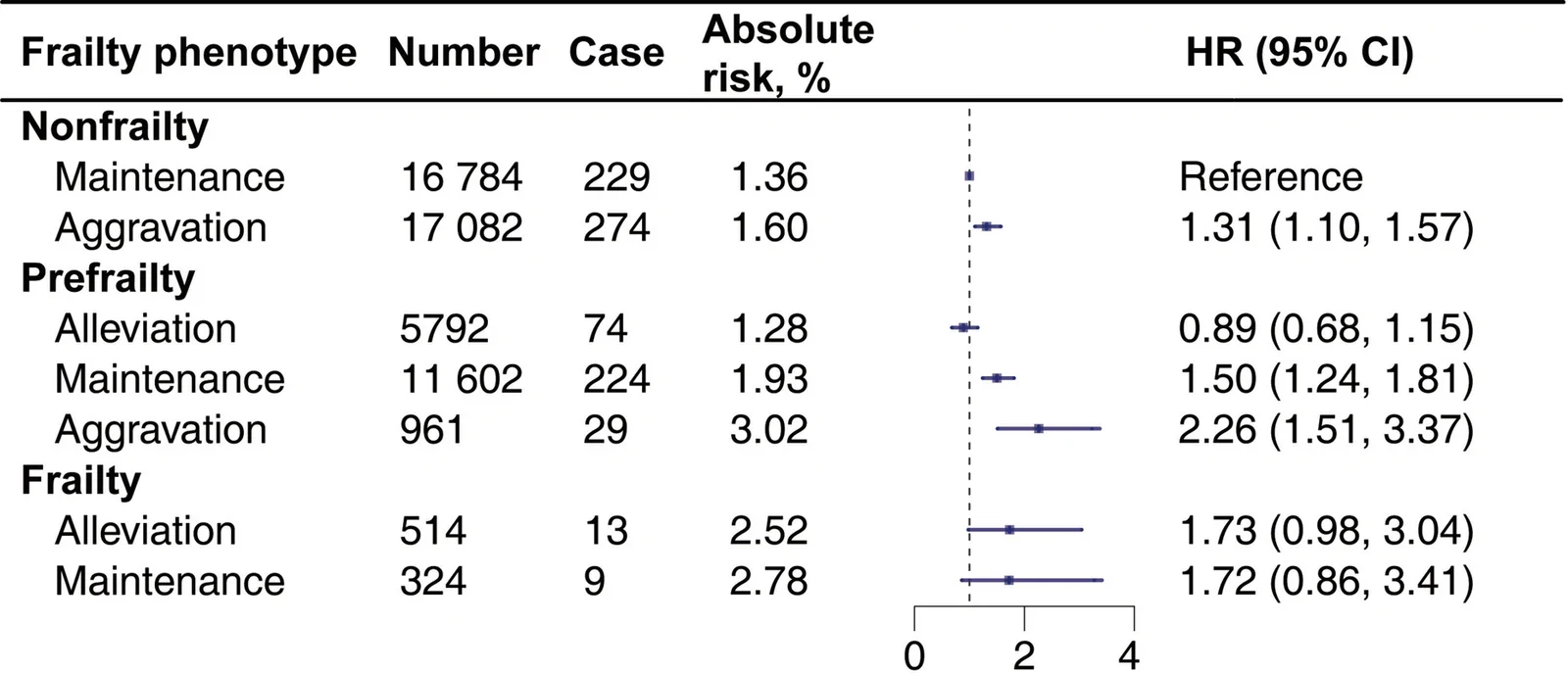
Associations of frailty trajectories with incident AMD. Models were adjusted for age, sex, ethnicity, Townsend deprivation index, education, body mass index, alcohol consumption, smoking status, diabetes, and hypertension. AMD, age-related macular degeneration; HR, hazard ratio; CI, confidence interval

Compared to the nonfrailty maintenance group, the risk of AMD was significantly increased for the nonfrailty aggravation (HR: 1.31; 95% CI: 1.10–1.57), prefrailty maintenance (HR: 1.50; 95% CI: 1.24–1.81), and prefrailty aggravation (HR: 2.26; 95% CI: 1.51–3.37) trajectories (Fig. 2). However, frailty maintenance was not significantly associated with AMD risk (HR: 1.72; 95% CI: 0.86–3.41; *P* = 0.123), while frailty alleviation showed borderline significance (HR: 1.73; 95% CI: 0.98–3.04; *P* = 0.057). Among all trajectory combinations, prefrailty aggravation demonstrated the highest AMD risk (HR: 2.26; 95% CI: 1.51–3.37). PAR analysis estimated that 24.0% (95% CI: 17.5–30.7) of incident AMD cases could theoretically be prevented if all participants maintained nonfrailty. Additionally, worsening frailty status among nonfrail and prefrail individuals, combined with persistent frailty in initially frail individuals, contributed to a PAR of 2.7% (95% CI: 0.1–5.4).

### Sensitivity analyses

In the sensitivity analyses, we evaluated associations between frailty and AMD risk using the FI, another common frailty assessment metric. Baseline frailty prevalence was higher when measured by FI (2.1%) compared to FP (1.6%). At the final assessment, however, frailty prevalence measured by FI (2.3%) was lower than that identified by FP (3.1%; Supplementary Tables 6–7). Each 0.01-point/year increase in FI progression (ΔFI) was associated with a 28% elevated AMD risk (HR: 1.28; 95% CI: 1.15–1.42; Supplementary Table 8). Analyses of baseline FI and longitudinal trajectories revealed that all frailty trajectory combinations significantly increased AMD risk compared to stable nonfrailty. Frailty maintenance (HR: 1.81; 95% CI: 1.13–2.90) and alleviation (HR: 1.75; 95% CI: 1.04–2.95) showed the highest AMD risks (Supplementary Table 8). A broadly comparable pattern was observed in combined analyses of FP and FI trajectories, even after excluding participants with less than 2-year follow-up, those in poor baseline health, or adjusting for HDL, LDL, triglycerides, AMD polygenic risk score, and modifying the covariates estimated in the final evaluation instead of at baseline (Supplementary Tables 9–12).

## Discussion

Our study demonstrates an association between progressive frailty trajectories and increased AMD risk. Each 1-point increase in frailty scores was associated with a 28% higher AMD risk. A dose–response relationship between FP alterations and AMD risk was observed, with a 0.1-point annual increase in FP scores being correlated with a 15% elevated AMD risk over a median 3.3-year follow-up. Additionally, maintaining a nonfrail status could prevent 24.0% of incident AMD cases, while mitigating frailty progression in nonfrail or prefrail individuals and preventing frailty persistence in frail individuals could reduce AMD incidence by 2.7%.

Emerging evidence highlights dynamic frailty trajectories as predictors of adverse health outcomes, including metabolic disorders [33] and mortality [34]; however, their association with AMD remains underexplored. In this longitudinal investigation using UK Biobank data, we identified accelerated frailty progression to be independently associated with an elevated risk of incident AMD, irrespective of baseline frailty status. A more pronounced association between frailty and AMD was observed in the trajectory analysis compared to the baseline assessment. This is likely because the trajectory analysis used a refined reference group of individuals who sustained a nonfrail status, whereas the single-timepoint baseline group was diluted by participants who developed frailty during follow-up, which attenuates the risk estimate. Analyses combining baseline and trajectory data showed a progressive increase in AMD risk associated with deteriorating frailty trajectories of maintenance and aggravation among those initially classified as nonfrail or prefrail. Interestingly, analyses based on the FI consistently demonstrated an elevated AMD risk even in cases of frailty improvement, suggesting that transient improvements in frailty status may not fully mitigate the systemic consequences of prior deficit accumulation. These results underscore the clinical imperative for implementing early, sustained interventions targeting frailty dynamics, even in populations demonstrating temporary trajectory improvements, to prevent irreversible systemic damage that may drive AMD pathogenesis.

The pathophysiological interplay between frailty and AMD may arise from multisystemic interactions involving shared aging-related mechanisms. Chronic inflammation, acting as a central driver, may lead to disruptions in the retinal pigment epithelial barrier function due to elevated pro-inflammatory factors in frailty states, thereby contributing to the development of AMD [16, 17]. Mitochondrial dysfunction, a key factor in frailty, coupled with increased oxidative stress, could further worsen damage to photoreceptor cells [18, 35]. Additionally, the cumulative deficits reflected by FI may exert lasting effects through epigenetic reprogramming; even when clinical indicators improve, altered DNA methylation patterns might persistently compromise antioxidant defense systems [36]. The convergence of these pathophysiological processes underscores the importance of early identification and precise management of shared aging-related pathways to disrupt the reciprocal cycle between systemic frailty and retinal degeneration.

Subgroup analyses revealed significant age-dependent interactions, indicating that the association between frailty and AMD is most prominent in participants under the age of 60. This observation is consistent with prior research studies [37–40], which suggest that frailty's predictive utility for disease outcomes diminishes with advancing age. The age-specific patterns suggest that frailty may accelerate the development of AMD in younger cohorts through mechanisms overlapping with accelerated aging, such as mitochondrial dysfunction, oxidative stress, and retinal senescence [22]. Conversely, in older individuals, the progression of AMD seems to be primarily influenced by age-related physiological deterioration. These results emphasize the potential role of frailty in the early pathogenesis of AMD and highlight clinical imperative for frailty assessment in middle-aged populations.

Our findings at the population level indicate that preventing the progression of frailty in nonfrail and prefrail individuals, as well as halting further decline in those already classified as frail, could potentially prevent around 2.7% of new AMD cases. This highlights the significance of frailty as a modifiable risk factor, emphasizing the need for its inclusion in public health strategies. Implementing evidence-based interventions, such as improving dietary patterns [41, 42], increasing physical activity [43], and regulating inflammatory pathways [17], may help counteract shared mechanisms that contribute to the development of both frailty and AMD. By addressing frailty as an indicator of accelerated aging, healthcare systems may concurrently mitigate physical disability and AMD-related disease burden, thereby promoting functional independence and visual health among aging populations.

Although our study offers new findings, there remain some limitations. First, investigating the associations between frailty progression trajectories and clinically staged AMD severity could provide valuable insights; however, the UK Biobank does not include data on AMD clinical stages. Second, the definition of frailty incorporates both objective measures and self-reported data, which may introduce reporting biases. Third, the UK Biobank cohort is susceptible to a "healthy volunteer" selection bias [44]. Participants included in the frailty trajectory analyses exhibited lower disease prevalence compared to the general population, potentially attenuating the observed associations between frailty and AMD. Nevertheless, existing evidence suggests that exposure-disease relationships derived from the UK Biobank retain generalizability despite limited population representativeness [44]. Fourth, some subgroup findings did not yield statistical significance in the sensitivity analysis, hinting at potential instability due to limited statistical power. Similarly, due to restricted sample sizes, our subgroup analyses could not evaluate the effects of baseline frailty status and its longitudinal trajectories on AMD incidence. Fifth, despite comprehensive adjustments for potential confounders, residual confounding cannot be fully excluded. Sixth, as an observational study, our findings do not establish causality definitively; therefore, the PARs should be interpreted with caution. Lastly, the predominance of White participants in our study may limit the generalizability of findings to other racial or ethnic groups.

In conclusion, our study reveals a significant association between prefrailty, frailty, and progressive frailty trajectories and an increased risk of AMD. These results underscore the clinical imperative for early frailty detection and systematic monitoring in midlife populations. Implementing targeted interventions to halt the progression of frailty could potentially alleviate the burden of AMD in aging populations worldwide. Future studies are needed to validate these associations across diverse populations and explore mechanistic correlations between frailty and the pathogenesis of AMD.

## Supplementary Information

Below is the link to the electronic supplementary material.

Supplementary file1 (DOCX 530 kb)

## Data Availability

All UK Biobank information is available online (https://www.ukbiobank.ac.uk/). Data access is available through applications. This research has been conducted using the UK Biobank Resource under Application Number 91320.
