# Supplementary material for: Dynamic frailty trajectories and risk of age-related macular degeneration: a prospective cohort study in UK Biobank
Source: GeroScience. 2026 Jan 2;48(6):7681–91. doi: 10.1007/s11357-025-02066-x (PMC13638876; doi:10.1007/s11357-025-02066-x)
Supplement: Supplementary file 1 [file 11357_2025_2066_MOESM1_ESM.docx]

**Supplementary Table 1.** Definition of frailty.

| Frailty  indicators | UK Biobank question | Field ID |
| --- | --- | --- |
| Weight loss | Question: Compared with one year ago, has your weight changed?   - Yes - lost weight=1; - No - weigh about the same=0; - Yes - gained weight=0; - Do not know=0; - Prefer not to answer=excluded | 2306 |
| Exhaustion | Question: Over the past two weeks, how often have you felt tired or had little energy?   - Not at all=0; - Several days=0; - More than half the days=1; - Nearly every day=1; - Do not know=0; - Prefer not to answer=excluded | 2080 |
| Walking speed | Question: How would you describe your usual walking pace?   - Slow pace=1; - Steady average pace=0; - Brisk pace=0; - None of the above/Prefer not to answer=excluded | 924 |
| Weakness | Grip strength was assessed isometrically using a calibrated J00105 hydraulic hand dynamometer (Lafayette Instrument Company, IN, USA), separately in the left and right arms. The average of the right and left measurement was used here, or the available one when either measurement is missing.  Cut-offs used to define low grip strength:   - Males:   BMI≤24 & grip strength≤29; 24.1≤BMI≤28 & grip strength≤30; BMI>28 & grip strength≤32   - Females:   BMI≤23 & grip strength≤17; 23.1≤BMI≤26 & grip strength≤17.3; 26.1≤BMI≤29 & grip strength≤18; BMI>29 & grip strength≤21 | 46; 47 |
| Physical activity | Question: In the last 4 weeks did you spend any time doing the following?   - Walking for pleasure =0; - Strenuous sports=0; - Light DIY (eg: pruning, watering the lawn): Frequency of once per week or less=1; Frequency of more than once per week=0; - Heavy DIY (eg: weeding, lawn mowing, carpentry, digging) =0; - Other exercises (eg: swimming, cycling, keep fit, bowling) =0; - None of the above=1; - Prefer not to answer=excluded | 6164;  1011 |

**Supplementary Table 2**. Questionnaire items from the baseline UK Biobank assessment used to compose the Frailty Index.

| 1. Glaucoma | 18. Diabetes | 35. Any cancer diagnosis |
| --- | --- | --- |
| 2. Cataracts | 19. Coronary heart disease | 36. Multiple cancers diagnosed |
| 3. Hearing difficulty | 20. Angina | 37. Chest pain |
| 4. Migraine | 21. Stroke | 38. Head and/or neck pain |
| 5. Dental problems | 22. High blood pressure | 39. Back pain |
| 6. Self-rated health | 23. Deep-vein thrombosis | 40. Stomach/abdominal pain |
| 7. Fatigue | 24. High cholesterol | 41. Hip pain |
| 8. Sleeplessness/insomnia | 25. Breathing (wheeze) | 42. Knee pain |
| 9. Depressed feelings | 26. Pneumonia | 43. Whole-body pain |
| 10. Self-described nervous  personality | 27. Chronic bronchitis/emphysema | 44. Facial pain |
| 11. Severe anxiety/panic attacks | 28. Asthma | 45. Hypothyroidism |
| 12. Loneliness | 29. Rheumatoid arthriti | 46. Gastric reflux |
| 13. Misery | 30. Osteoarthritis | 47. Hiatus hernia |
| 14. Long-standing illness or disability | 31. Gout | 48. Gall stones |
| 15. Falls | 32. Osteoporosis | 49. Diverticulitis |
| 16. Fractures/broken bones | 33. Hayfever, allergic rhinitis or eczema |  |
| 17. Sciatica | 34. Psoriasis |  |

**Supplementary Table 3**. The resource and definition of the selected covariates (except for age, and sex).

| **Covariates** | **UK BioBank**  **Code** | **Description** | **Type** | **Category** |
| --- | --- | --- | --- | --- |
| Ethnicity | 21000 | Ethnic background | Categorical | Whites;  Non-whites |
| Townsend deprivation index | 189 | The existing variable 'Townsend area deprivation index' (189) served as an  indicator of socioeconomic status, with higher scores indicating greater  socioeconomic deprivation. | Continuous | NA |
| Education | 6138 | Answers were from ACE touchscreen question "Which of the following qualifications do you have? (You can select more than one)". The existing variable ' Qualifications' (6138) was applied. | Categorical | NVQ/CSE/A levels/others;  College or university degree;  None |
| Body mass index | 21001 | Body mass index was calculated as weight (kg) divided by squared height (m^2^)). | Continuous | NA |
| Smoking status | 20116 | The existing variable 'smoking status' (20116) was applied. | Categorical | Never;  Previous smoker;  Current smoker |
| Alcohol consumption | 20117 | The existing variable 'Alcohol drinker status ' (20117) was applied. | Categorical | Never;  Previous drinker;  Current drinker |
| Diabetes | 2443, 2976, 6153, 6177, 20002 | Diabetes status was defined as history of diabetes, glucose ≥7.0 mmol/L, or HbA1c ≥6.5%. | Categorical | Yes;  No |
| Hypertension | 2966, 6150, 6153, 6177, 20002 | Hypertension was defined as history of hypertension. | Categorical | Yes;  No |

For further information, please refer to the UK Biobank data showcase https://biobank.ndph.ox.ac.uk/showcase/search.cgi

**Supplementary Table 4.** Characteristics of participants in the baseline and the trajectories analyses with frailty phenotype.

|  |  | Baseline assessment ^b^  (n=462,573) | Trajectories analysis ^c^ (n=53,059) | |
| --- | --- | --- | --- | --- |
| Characteristics ^a^ | |  | Baseline assessment | Trajectories assessment |
| Age, mean (SD), years | | 56.38 ± 8.1 | 55.44 ± 7.6 | 63.47 ± 7.7 |
| Men, n (%) | | 209,727 (45.3) | 25,667 (48.4) | |
| White ethnicity, n (%) | | 438,298 (94.8) | 51,517 (97.1) | |
| University or college degree, n (%) | | 153,297 (33.1) | 24,094 (45.4) | |
| Townsend deprivation index, mean (SD) | | -1.36± 3.1 | -1.92± 2.7 | |
| Alcohol consumption, n (%) | | |  |  |
|  | Current | 427,486 (92.4) | 50,497 (95.2) | 49,526 (93.3) |
|  | Former | 15,839 (3.4) | 1183 (2.2) | 1768 (3.3) |
|  | Never | 19,248 (4.2) | 1381 (2.6) | 1729 (3.3) |
| Smoking status, n (%) | |  |  |  |
|  | Current | 47,474 (10.3) | 3378 (6.4) | 2017 (3.8) |
|  | Former | 159,977 (34.6) | 17,662 (33.3) | 18,107 (34.1) |
|  | Never | 255,122 (55.2) | 32,021 (60.3) | 32,887 (62.0) |
| Body mass index, mean (SD), kg/m^2^ | | 27.37 ± 4.7 | 26.69 ± 4.3 | 26.66 ± 4.5 |
| Diabetes, n (%) | | 21,679 (4.7) | 1639 (3.1) | 3521 (6.6) |
| Hypertension, n (%) | | 120,302 (26.0) | 11,999 (22.6) | 18,565 (35.0) |
| Frailty phenotype | |  |  |  |
|  | Nonfrailty, n (%) | 255,079 (55.1) | 33,868 (63.8) | 22,628 (42.6) |
|  | Prefrailty, n (%) | 188,948 (40.8) | 18,355 (34.6) | 28,803 (54.3) |
|  | Frailty, n (%) | 18,546 (4.0) | 838 (1.6) | 1630 (3.1) |

^a^ Continuous variables are presented as the means (standard deviations (SDs)), and categorical variables are presented as the numbers (percentages).

^b^ Baseline assessment refers to the single-timepoint frailty measurement at enrollment.

^c^ Trajectories assessment refers to the longitudinal analysis of frailty changes across follow-up measurements in the subcohort with repeated data.

**Supplementary Table 5.** Individual components of frailty and their association with incident AMD.

| Frailty component | HR (95% CI) |  |  |  |
| --- | --- | --- | --- | --- |
|  | Model 1 | Model 2 | Model 3 | Model 4 |
| Weight loss | 1.13 (1.08, 1.18) | 1.11 (1.06, 1.16) | 1.05 (1.00, 1.10) | 1.05 (1.00, 1.10) |
| Exhaustion | 1.28 (1.21, 1.34) | 1.19 (1.13, 1.26) | 1.15 (1.09, 1.22) | 1.11 (1.06, 1.18) |
| Low physical activity | 1.22 (1.16, 1.29) | 1.13 (1.07, 1.19) | 1.10 (1.04, 1.16) | 1.05 (0.99, 1.11) |
| Weakness | 1.27 (1.22, 1.32) | 1.20 (1.15, 1.25) | 1.17 (1.12, 1.22) | 1.15 (1.10, 1.20) |
| Slow walking speed | 1.41 (1.34, 1.49) | 1.27 (1.20, 1.35) | 1.20 (1.14, 1.27) | 1.14 (1.07, 1.21) |

Model 1: adjusted for age and sex.

Model 2: Model 1 variables plus ethnicity, Townsend deprivation index, education, body mass index, alcohol consumption, and smoking status.

Model 3: Model 2 variables plus diabetes, and hypertension.

Model 4: Model 3 variables plus 5 frailty components (mutual adjustment).

Abbreviations: AMD, age-related macular degeneration; HR, hazard ratio; CI, confidence interval.

**Supplementary Table 6.** Characteristics of participants in the baseline and the trajectories analyses with frailty index.

|  |  | Baseline assessment ^b^  (n=458,224) | Trajectories analysis ^c^ (n=55,827) | |
| --- | --- | --- | --- | --- |
| Characteristics ^a^ | |  | Baseline assessment | Trajectories assessment |
| Age, mean (SD), years | | 56.38 ± 8.1 | 55.61 ± 7.6 | 63.66 ± 7.7 |
| Men, n (%) | | 208,304 (45.5) | 28,800 (51.6) | |
| White ethnicity, n (%) | | 433,898 (94.7) | 54,169 (97.0) | |
| University or college degree, n (%) | | 152,250 (33.2) | 25,345 (45.4) | |
| Townsend deprivation index, mean (SD) | | -1.37 ± 3.1 | -1.91 ± 2.7 | |
| Alcohol consumption, n (%) | | |  |  |
|  | Current | 423,317 (92.4) | 53,100 (95.1) | 52,067 (93.3） |
|  | Former | 15,681 (3.4) | 1262 (2.3) | 1874 (3.4) |
|  | Never | 19,226 (4.2) | 1467 (2.6) | 1841 (3.3) |
| Smoking status, n (%) | |  |  |  |
|  | Current | 46,809 (10.2) | 3512 (6.3) | 2081 (3.7) |
|  | Former | 158,363 (34.6) | 18,745 (33.6) | 19,094 (34.2) |
|  | Never | 253,052 (55.2) | 33,572 (60.1) | 34,464 (61.7) |
| Body mass index, mean (SD), kg/m^2^ | | 27.36 ± 4.7 | 26.70 ± 4.3 | 26.66 ± 4.5 |
| Diabetes, n (%) | | 21,477 (4.7) | 1779 (3.2) | 3781 (6.8) |
| Hypertension, n (%) | | 11,8945 (26.0) | 12,767 (22.9) | 19,693 (35.3) |
| Frailty inidex | |  |  |  |
|  | Nonfrailty, n (%) | 238,980 (52.2) | 33,496 (60.0) | 33,067 (59.2) |
|  | Prefrailty, n (%) | 199,146 (43.5) | 21,133 (37.9) | 21,461 (39.4) |
|  | Frailty, n (%) | 20,098 (4.4) | 1200 (2.1) | 1301 (2.3) |

^a^ Continuous variables are presented as the means (standard deviations (SDs)), and categorical variables are presented as the numbers (percentages).

^b^ Baseline assessment refers to the single-timepoint frailty measurement at enrollment.

^c^ Trajectories assessment refers to the longitudinal analysis of frailty changes across follow-up measurements in the subcohort with repeated data.

**Supplementary Table 7.** Characteristics of participants in the baseline and the trajectories assessment according to frailty index.

|  |  | Baseline assessment ^b^ | | | Trajectories assessment ^c^ | | |
| --- | --- | --- | --- | --- | --- | --- | --- |
| Characteristics ^a^ | | Nonfrailty | Prefrailty | Frailty | Nonfrailty | Prefrailty | Frailty |
| Sample size, n | | 238,980 | 199,146 | 20,098 | 33,066 | 21,460 | 1301 |
| Age, mean (SD), years | | 55.59 ± 8.1 | 57.20 ± 8.0 | 57.80 ± 7.6 | 55.07 ± 7.5 | 56.43 ± 7.6 | 55.96 ± 7.6 |
| Men, n (%) | | 113,350 (47.4) | 86,254 (43.3) | 8700 (43.3) | 16,328 (49.4) | 10,110 (47.1) | 591 (45.4) |
| White ethnicity, n (%) | | 227,024 (95.0) | 188,207 (94.5) | 18,667 (92.9) | 32,067 (97.0) | 20,850 (97.2) | 1252 (96.2) |
| University or college degree, n (%) | | 90,363 (37.8) | 58,181 (29.2) | 3706 (18.4) | 15,951 (48.2) | 9001 (41.9) | 393 (30.2) |
| Townsend deprivation index, mean (SD) | | -1.70 ± 2.9 | -1.15 ± 3.1 | 0.35 ± 3.6 | -2.04 ± 2.7 | -1.78 ± 2.8 | -0.78 ± 3.2 |
| Alcohol consumption, n (%) | | |  |  |  |  |  |
|  | Current | 224,711 (94.0) | 181,945 (91.4) | 16,661 (82.9) | 31,649 (95.7) | 20,290 (94.5) | 1161 (89.2) |
|  | Former | 5430 (2.3） | 8263 (4.1) | 1988 (9.9) | 574 (1.7) | 601 (2.8) | 87 (6.7) |
|  | Never | 8839 (3.7) | 8938 (4.5) | 1449 (7.2) | 844 (2.6) | 570 (2.7) | 53 (4.1) |
| Smoking status, n (%) | |  |  |  |  |  |  |
|  | Current | 19,960 (8.4) | 22,793 (11.4) | 4056 (20.2) | 1811 (5.5) | 1563 (7.3) | 138 (10.6) |
|  | Former | 75,833 (31.7) | 74,611 (37.5) | 7919 (39.4) | 10,214 (30.9) | 7989 (37.2) | 542 (41.7) |
|  | Never | 143,187 (59.9) | 101,742 (51.1) | 8123 (40.4) | 21,042 (63.6) | 11,909 (55.5) | 621 (47.7) |
| Body mass index, mean (SD), kg/m^2^ | | 26.58 ± 4.2 | 28.01 ± 5.0 | 30.36 ± 6.1 | 26.12 ± 3.9 | 27.39 ± 4.6 | 29.80 ± 5.8 |
| Diabetes, n (%) | | 3766 (1.6) | 14,062 (7.1) | 3649 (18.2) | 401 (1.2) | 1204 (5.6) | 174 (13.4) |
| Hypertension, n (%) | | 37,619 (15.7) | 69,918 (35.1) | 11,408 (56.8) | 5277 (16.0) | 6856 (31.9) | 634 (48.7) |

^a^ Characteristics are presented for all participants at the time of their enrollment.

^b^ Baseline assessment refers to the frailty measurement at enrollment.

^c^ Trajectories assessment refers to the longitudinal analysis of frailty changes across follow-up measurements in the subcohort with repeated data.

**Supplementary Table 8**. Association of baseline and long-term changes in frailty with incident AMD according to frailty index.

| Frailty index | | Sample size | Case | Absolute risk, % | HR (95% CI) |
| --- | --- | --- | --- | --- | --- |
| **Baseline assessment** | |  |  |  |  |
|  | Nonfrailty | 238,980 | 5605 | 2.35 | Reference |
|  | Prefrailty | 199,146 | 6741 | 3.38 | 1.17 (1.13, 1.22) |
|  | Frailty | 20,098 | 950 | 4.73 | 1.48 (1.38, 1.59) |
|  | Per 0.1 point increase |  |  |  | 1.26 (1.22, 1.30) |
| **Trajectories assessment** | |  |  |  |  |
|  | Nonfrailty | 33,066 | 440 | 1.33 | Reference |
|  | Prefrailty | 21,460 | 441 | 2.05 | 1.44 (1.26, 1.66) |
|  | Frailty | 1301 | 35 | 2.69 | 1.51 (1.08, 2.22) |
|  | Per 0.1 point increase |  |  |  | 1.52 (1.34, 1.71) |
| ΔFI per 0.01 point/year increase | | |  |  | 1.28 (1.15, 1.42) |
|  | | |  |  |  |
| **Frailty change** | |  |  |  |  |
| Nonfrailty | |  |  |  |  |
|  | Maintenance | 26,554 | 331 | 1.25 | Reference |
|  | Aggravation | 6942 | 121 | 1.74 | 1.43 (1.16, 1.76) |
| Prefrailty | |  |  |  |  |
|  | Alleviation | 6473 | 108 | 1.67 | 1.25 (1.00, 1.56) |
|  | Maintenance | 13,901 | 302 | 2.17 | 1.56 (1.33, 1.84) |
|  | Aggravation | 757 | 19 | 2.51 | 1.67 (1.04, 2.67) |
| Frailty | |  |  |  |  |
|  | Alleviation | 693 | 19 | 2.74 | 1.81 (1.13, 2.90) |
|  | Maintenance | 507 | 16 | 3.16 | 1.75 (1.04, 2.95) |

Model was adjusted for age, sex, ethnicity, Townsend deprivation index, education, body mass index, alcohol consumption, smoking status, diabetes, and hypertension. For trajectory analysis in frailty (ΔFI), the baseline frailty index was further adjusted in the Cox regression models. AMD, age-related macular degeneration; HR, hazard ratio; CI, confidence interval.

**Supplementary Table 9.** Associations of frailty trajectories with incident AMD (participants who had follow-up time less than 2 years were excluded).

|  |  | Number | Case | Absolute risk, % | HR (95% CI) |
| --- | --- | --- | --- | --- | --- |
| **Frailty phenotype** | |  |  |  |  |
| Nonfrailty | |  |  |  |  |
|  | Maintenance | 12,361 | 166 | 1.34 | Reference |
|  | Aggravation | 12,372 | 185 | 1.50 | 1.22 (0.99, 1.52) |
| Prefrailty | |  |  |  |  |
|  | Alleviation | 4197 | 54 | 1.29 | 0.91 (0.67, 1.24) |
|  | Maintenance | 8658 | 173 | 2.00 | 1.54 (1.23, 1.92) |
|  | Aggravation | 720 | 25 | 3.47 | 2.45 (1.58, 3.80) |
| Frailty | |  |  |  |  |
|  | Alleviation | 395 | 4 | 1.01 | 0.68 (0.25, 1.84) |
|  | Maintenance | 271 | 8 | 2.94 | 1.77 (0.85, 3.68) |
|  |  |  |  |  |  |
| **Frailty index** | |  |  |  |  |
| Nonfrailty | |  |  |  |  |
|  | Maintenance | 19,119 | 234 | 1.22 | Reference |
|  | Aggravation | 4897 | 84 | 1.72 | 1.39 (1.08, 1.79) |
| Prefrailty | |  |  |  |  |
|  | Alleviation | 4632 | 69 | 1.49 | 1.14 (0.87, 1.49) |
|  | Maintenance | 10,039 | 207 | 2.06 | 1.40 (1.15, 1.71) |
|  | Aggravation | 499 | 14 | 2.81 | 1.66 (0.96, 2.88) |
| Frailty | |  |  |  |  |
|  | Alleviation | 404 | 13 | 3.22 | 2.22 (1.26, 3.91) |
|  | Maintenance | 242 | 12 | 4.96 | 2.95 (1.62, 5.35) |

Model was adjusted for age, sex, ethnicity, Townsend deprivation index, education, body mass index, alcohol consumption, smoking status, diabetes, and hypertension. AMD, age-related macular degeneration; HR, hazard ratio; CI, confidence interval.

**Supplementary Table 10.** Associations of frailty trajectories with incident AMD (participants with self-reported poor health at baseline were excluded).

|  |  | Number | Case | Absolute risk, % | HR (95% CI) |
| --- | --- | --- | --- | --- | --- |
| **Frailty phenotype** | |  |  |  |  |
| Nonfrailty | |  |  |  |  |
|  | Maintenance | 16,705 | 228 | 1.36 | Reference |
|  | Aggravation | 16,958 | 273 | 1.61 | 1.31 (1.09, 1.57) |
| Prefrailty | |  |  |  |  |
|  | Alleviation | 5687 | 74 | 1.30 | 0.90 (0.69, 1.18) |
|  | Maintenance | 11,231 | 218 | 1.94 | 1.51 (1.24, 1.83) |
|  | Aggravation | 842 | 26 | 3.09 | 2.36 (1.55, 3.58) |
| Frailty | |  |  |  |  |
|  | Alleviation | 401 | 10 | 2.49 | 1.74 (0.92, 3.30) |
|  | Maintenance | 182 | 4 | 2.20 | 1.53 (0.56, 4.15) |
|  |  |  |  |  |  |
| **Frailty index** | |  |  |  |  |
| Nonfrailty | |  |  |  |  |
|  | Maintenance | 26,520 | 331 | 1.25 | Reference |
|  | Aggravation | 6917 | 121 | 1.75 | 1.43 (1.16, 1.76) |
| Prefrailty | |  |  |  |  |
|  | Alleviation | 6370 | 105 | 1.65 | 1.24 (0.99, 1.55) |
|  | Maintenance | 13,369 | 289 | 2.16 | 1.55 (1.31, 1.83) |
|  | Aggravation | 667 | 19 | 2.85 | 1.88 (1.18, 3.01) |
| Frailty | |  |  |  |  |
|  | Alleviation | 545 | 14 | 2.57 | 1.85 (1.07, 3.17) |
|  | Maintenance | 284 | 14 | 4.93 | 3.04 (1.76, 5.25) |

Model was adjusted for age, sex, ethnicity, Townsend deprivation index, education, body mass index, alcohol consumption, smoking status, diabetes, and hypertension. AMD, age-related macular degeneration; HR, hazard ratio; CI, confidence interval.

**Supplementary Table 11.** Associations of frailty trajectories with incident AMD with further adjustments.

|  |  | Model 3 + HDL, LDL, triglycerides ^a^ | Model 3 + AMD polygenic risk score ^b^ |
| --- | --- | --- | --- |
| **Frailty phenotype** | |  |  |
| Nonfrailty | |  |  |
|  | Maintenance | Reference | Reference |
|  | Aggravation | 1.37 (1.13, 1.65) | 1.33 (1.11, 1.60) |
| Prefrailty | |  |  |
|  | Alleviation | 0.83 (0.62, 1.11) | 0.89 (0.68, 1.16) |
|  | Maintenance | 1.51 (1.23, 1.85) | 1.52 (1.25, 1.84) |
|  | Aggravation | 2.45 (1.60, 3.74) | 2.29 (1.54, 3.42) |
| Frailty | |  |  |
|  | Alleviation | 1.81 (0.98, 3.34) | 1.71 (0.97, 3.01) |
|  | Maintenance | 1.56 (0.72, 3.39) | 1.76 (0.89, 3.50) |
|  |  |  |  |
| **Frailty index** | |  |  |
| Nonfrailty | |  |  |
|  | Maintenance | Reference | Reference |
|  | Aggravation | 1.47 (1.18, 1.84) | 1.44 (1.16, 1.78) |
| Prefrailty | |  |  |
|  | Alleviation | 1.28 (1.01, 1.63) | 1.26 (1.01, 1.57) |
|  | Maintenance | 1.61 (1.35, 1.92) | 1.60 (1.35, 1.89) |
|  | Aggravation | 1.93 (1.18, 3.18) | 1.78 (1.11, 2.84) |
| Frailty | |  |  |
|  | Alleviation | 1.48 (0.84, 2.60) | 1.86 (1.16, 2.99) |
|  | Maintenance | 2.05 (1.19, 3.54) | 1.85 (1.10, 3.12) |

^a^ Model was adjusted for age, sex, ethnicity, Townsend deprivation index, education, body mass index, alcohol consumption, smoking status, diabetes, hypertension, plasma high-density lipoprotein cholesterol, plasma low-density lipoprotein cholesterol, and plasma triglycerides.

^b^ Model was adjusted for age, sex, ethnicity, Townsend deprivation index, education, body mass index, alcohol consumption, smoking status, diabetes, hypertension, and AMD polygenic risk score.

AMD, age-related macular degeneration; HDL, high-density lipoprotein cholesterol; LDL, low-density lipoprotein cholesterol; HR, hazard ratio; CI, confidence interval.

**Supplementary Table 12.** Associations of frailty trajectories with incident AMD (model adjusted for covariates in the final assessment).

|  |  | Number | Case | Absolute risk, % | HR (95% CI) |
| --- | --- | --- | --- | --- | --- |
| **Frailty phenotype** | |  |  |  |  |
| Nonfrailty | |  |  |  |  |
|  | Maintenance | 16,784 | 229 | 1.36 | Reference |
|  | Aggravation | 17,082 | 274 | 1.60 | 1.28 (1.07, 1.53) |
| Prefrailty | |  |  |  |  |
|  | Alleviation | 5792 | 74 | 1.28 | 0.89 (0.68, 1.15) |
|  | Maintenance | 11,602 | 224 | 1.93 | 1.48 (1.22, 1.79)) |
|  | Aggravation | 961 | 29 | 3.02 | 2.17 (1.46, 3.23) |
| Frailty | |  |  |  |  |
|  | Alleviation | 514 | 13 | 2.53 | 1.66 (0.94, 2.92) |
|  | Maintenance | 324 | 9 | 2.78 | 1.75 (0.88, 3.45) |
|  |  |  |  |  |  |
| **Frailty index** | |  |  |  |  |
| Nonfrailty | |  |  |  |  |
|  | Maintenance | 26,554 | 331 | 1.25 | Reference |
|  | Aggravation | 6942 | 121 | 1.74 | 1.29 (1.04, 1.60) |
| Prefrailty | |  |  |  |  |
|  | Alleviation | 6473 | 108 | 1.67 | 1.22 (0.98, 1.53) |
|  | Maintenance | 13,901 | 302 | 2.17 | 1.48 (1.26, 1.75) |
|  | Aggravation | 757 | 19 | 2.51 | 1.51 (0.94, 2.42) |
| Frailty | |  |  |  |  |
|  | Alleviation | 693 | 19 | 2.74 | 1.65 (1.02, 2.68) |
|  | Maintenance | 507 | 16 | 3.16 | 1.75 (1.04, 2.94) |

Model was adjusted for age, sex, ethnicity, Townsend deprivation index, education, body mass index, alcohol consumption, smoking status, diabetes, and hypertension. AMD, age-related macular degeneration; HR, hazard ratio; CI, confidence interval.

**Supplementary Figure 1.** Participant flow diagram.

**Supplementary Figure 2.** Definition and corresponding time for the assessments and analyses in current study.

**Supplementary Figure 3.** **Dose-response relationship between ΔFP and incident AMD.** Model was adjusted for age, sex, ethnicity, Townsend deprivation index, education, body mass index, alcohol consumption, smoking status, diabetes, hypertension and baseline frailty score. HR, hazard ratio; CI, confidence interval; AMD, age-related macular degeneration.
